# Significance of Postprocedural Contrast Medium Injection after CT-Guided Abscess Drainage

**DOI:** 10.3390/tomography9040114

**Published:** 2023-07-27

**Authors:** Holger Goessmann, Stephan Schleder, Christian Stroszczynski, Andreas G. Schreyer

**Affiliations:** 1 Department of Radiology, University Hospital Leipzig, 04103 Leipzig, Germany; holger.goessmann@medizin.uni-leipzig.de; 2 Department of Diagnostic and Interventional Radiology, Merciful Brothers Hospital St. Elisabeth, 94315 Straubing, Germany; stephan.schleder@klinikum-straubing.de; 3 Department of Radiology, University Hospital Regensburg, 93053 Regensburg, Germany; christian.stroszczynski@klinik.uni-regensburg.de; 4 Department of Diagnostic and Interventional Radiology, University Hospital Brandenburg, Brandenburg Medical School Theodor Fontane, 14770 Brandenburg, Germany

**Keywords:** computed tomography, drain, systemic inflammatory response syndrome (SIRS), post-surgical complications, intervention

## Abstract

The aim was to evaluate the additive clinical value of an additional post-procedural control-scan after CT-guided percutaneous abscess drainage (PAD) placement with contrast medium (CM) via the newly placed drain. All CT-guided PADs during a 33-month period were retrospectively analyzed. We analyzed two subgroups, containing patients with and without surgery before intervention. Additionally, radiological records were reevaluated, concerning severe inflammatory response syndrome (SIRS) during the intervention. A total of 499 drainages were placed under CT-guidance in 352 patients. A total of 197 drainages were flushed with CM directly after the intervention, and 51 (26%) showed an additional significant finding. An immediate change of therapy was found in 19 cases (9%). The subgroup that underwent surgery (120 CM-drainages; 32 (27%) additional findings; 13 (11%) immediate changes of therapy) showed no statistically significant difference compared to the subgroup without surgery (77 CM-drainages; 19 (25%) additional findings; 5 (6%) immediate changes of therapy). SIRS occurred in 2 of the 197 flushed drainages (1%) after CM application. An additional scan with CM injection via the newly placed drain revealed clinically significant additional information in almost 26% of the drainages reviewed in this study. In 9% of the cases this information led to an immediate change of therapy. Risks for SIRS are low.

## 1. Introduction

Fluid collections and abscesses are common complications in infectious and/or postoperative patients with a high rate of mortality when untreated [1]. Ever since the 1980s, image-guided percutaneous drain placement has been established as the therapy of choice since it provides a quicker and less traumatic treatment than surgery. This method has been proven to be effective and safe [2]. It can be used to temporize a patient who will ultimately need surgery and can also be a definitive treatment for thoraco-abdominal fluid collections. Those fluid collections might originate from a perforated intestine, anastomotic leakages, inflammatory diseases of organs or bowels [3] or could be secondary to bacteremia [4].

A precise and fast diagnosis is a prerequisite for an appropriate and timely therapy. In particular, CT with augmented visualization options such as MIP (maximum intensity projection) reconstructions allow a stable method to depict even small amounts of contrast media or small air collections. Therefore, CT and MRI can provide crucial diagnostic information for consecutive drainage therapy. Despite the variety of imaging modalities available for detecting fluid collections, image-guided drainage placement is mostly performed under sonographic or CT guidance. While sonography is often utilized as guidance for fluid collections that are located superficially [5] and is the modality of choice for pediatric interventions [6], CT guidance is used mostly for accessing fluid collections deeper within the thorax, abdomen or pelvis, especially when using a transgluteal approach [3,7,8]. Even though percutaneous abscess drainage (PAD) is performed routinely nowadays, there is no clear recommendation as to whether or not PAD should be flushed with diluted contrast medium (CM) after CT-guided placement to gain additional information about the origin or possible co-factors of the fluid collection. Water soluble CM is routinely used to confirm anastomotic leakage after colorectal [9], biliary [10] or gastroesophageal surgery [11] with an endoluminal approach, and a CM inflow from an extraluminal drain has been proven to be feasible [12].

However, to the best of our knowledge, only one publication has ever emphasized the added clinical value of postprocedural contrast medium flushing of the newly placed CT-guided drainages [13]. However, some recent publications have highlighted the feasibility and clinical value of intracavitary injection of the ultrasound contrast medium [14,15]. Nevertheless, sonography is strongly dependent on the examiner’s experience and the patient’s condition and physiognomy. Especially in obese patients, sonography is frequently inconclusive [16].

Postprocedural flushing of newly placed drainages in CT is probably not routinely performed since some authors have reported systemic inflammatory response syndrome (SIRS) after performing fistulography [17,18]. However, in other drainages, like percutaneous transluminal biliary drainage (PTBD), cholangitis and sepsis can occur even without CM injection and adequate antibiotic coverage [19,20].

Therefore, the aim of this study was to evaluate the clinical value of an additional post-procedural control scan after CT-guided drainage placement with CM via the newly placed drain, as well as to analyze the probability for developing a systemic inflammatory response syndrome (SIRS) during the postprocedural CM injection.

## 2. Materials and Methods

### 2.1. Patients

**Figure 1:**
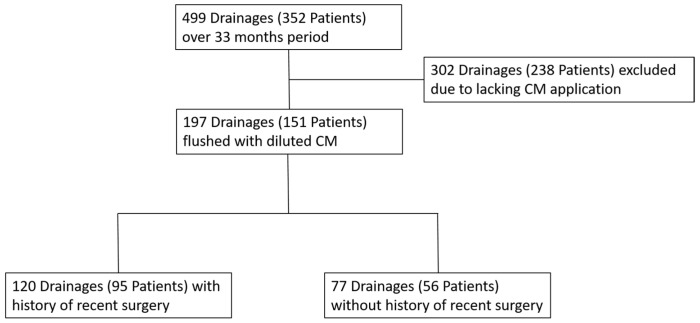
Patient selection over a 33-month period. Patients with drainages flushed with diluted contrast medium (CM) were selected for further evaluation.

We retrospectively evaluated all patients who underwent a CT-guided percutaneous chest or abdominal drainage (PAD) for suspected abscesses in our tertiary care hospital during a 33-month period between January 2014 and September 2016 (retrospective observational). Patients were identified using a full database query in the radiological information system (RIS, Nexus.medRIS, Version 8.42, Nexus, Villingen-Schwenningen, Germany). Based on this query, the radiological examinations in the PACS (picture archiving and communicating system), as well as the documentation in the RIS, were reviewed regarding a postprocedural contrast flush of the newly placed drain with a consecutive CT scan of the region of interest. The decision as to whether the drainage was flushed with diluted CM or not was performed at the preference of the interventional radiologist. Drainages which were not flushed with CM were excluded. Additionally, all drainages which were not intended to drain a clinically relevant fluid collection, such as ascites drains in preparation for further radiological interventions (e.g., percutaneous ablations), were excluded (Figure 1). Relevant clinical data concerning the patient’s history were acquired from RIS and HIS (hospital information system; SAP-R/3 IS-H i.s.h.med).

**Figure 2:**
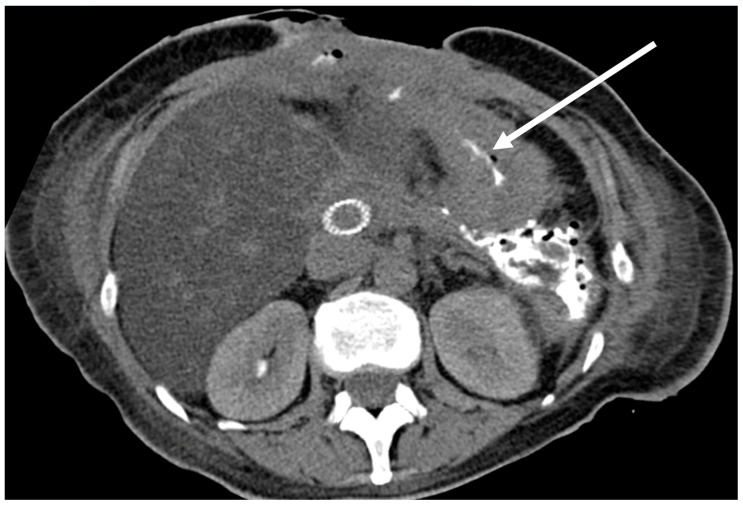
Patient with necrotizing pancreatitis and drainage of the fluid collection in the former region of the spleen. The post-procedural CM-flush showed CM in the gastric cavum (arrow), which did not result in a change of therapy (additional imaging finding).

**Figure 3:**
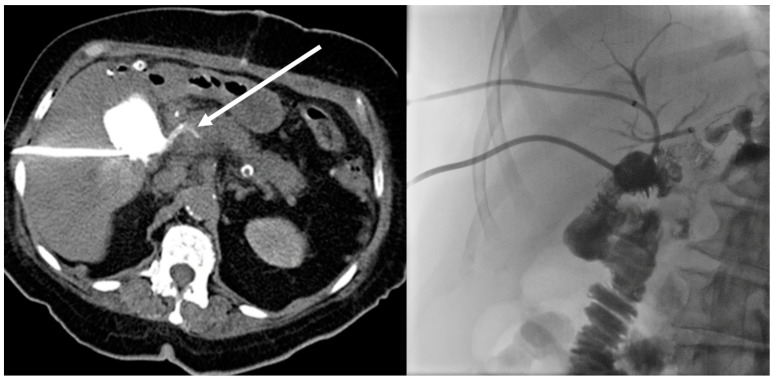
Patient with bilioma after Whipple procedure. The injected CM led to contrast in the common biliary duct (arrow). That proved an insufficient bilio-digestive anastomosis, leading to PTBD as an immediate change of therapy.

**Figure 4:**
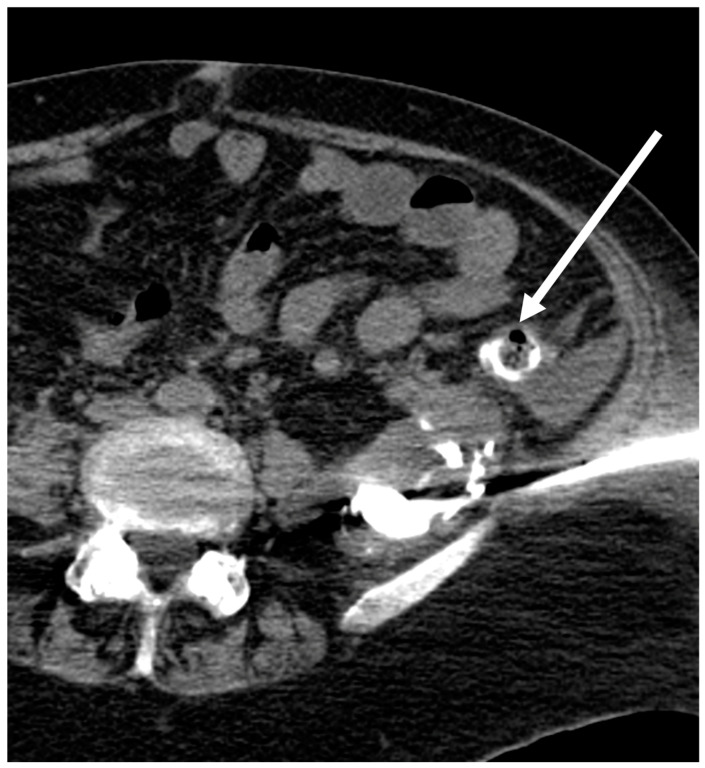
Patient with necrotizing pancreatitis. After CM injection via the newly placed drain, a fistula to the descending colon (arrow) was proven. The patient subsequently underwent right hemicolectomy.

The remaining drainages were evaluated regarding additional information based on the post-procedural scan. Therefore, two levels of additional information were defined. An additional imaging finding was stated when the injected CM communicated with anatomical structures or implanted medical devices that were likely to be the source of or an additional co-factor to the fluid collection (Figure 2). If that finding led to an immediate therapeutic measure, such as a change in medication or an endoscopic or surgical procedure, a change in therapy was stated (Figure 3 and Figure 4).

Radiological records were additionally checked for the occurrence of SIRS within the first 30 min after drainage placement. To estimate the additional radiation dose of the post-procedural CT scan, the dose length product (DLP) of the additional scan was acquired based on the examination protocol in the PACS. 

According to the independent ethics committee at our institution, an ethical vote for this retrospective evaluation was not necessary. All patients were treated according to the Declaration of Helsinki.

### 2.2. Drainage Procedure

All procedures were performed using a 16-line MD-CT (multidetector CT) scanner (Somatom 16; Siemens Health Care, Erlangen, Germany) under local anesthesia. Additional intravenous analgesic sedation was administered at the discretion of the radiologist, who performed the PAD. After selecting a suitable puncture site, the percutaneous drainage was placed inside the collecting fluid using the Seldinger technique. Drainages used in this retrospective study ranged from 6 to 24 French and were provided from different manufacturers (e.g., Cook Medical, Bloomington, IN, USA or Pflugbeil medizinische Instrument, Zorneding, Germany). The most commonly used drainage was the 8.5 F Dawson Mueller Drainage Catheter manufactured by Cook Medical (*n* = 194). After successful placement, the obtained liquid was preserved in a syringe for further lab workup. Thereafter, the drain was flushed with iodinated CM (Imeron 300, Bracco Diagnostics, Konstanz, Germany), usually diluted in the ratio of 1:4 with isotonic saline solution to reduce beam hardening artifacts. The volume of the injected CM solution was chosen by the performing radiologist individually to meet the requirement of the fluid collection. The drainage was flushed back and forth with the diluted CM carefully to achieve optimal distribution of the CM within the fluid collection. The length of the postprocedural scan was chosen according to the extent of the individual fluid collection based on the initial CT scan. All CT scans were performed as full dose scans (120 kV).

### 2.3. Subgroup Selection

The drainages included in this study were further divided into two subgroups. If a patient had undergone surgery or an interventional procedure of the thorax or the abdomen within the last 30 days, suitable to be correlated with the fluid collection, the patient was classified into the “surgery group”. This selection was maintained even if the patient was discharged from our hospital and readmitted with the newly developed fluid collection. If no such procedure had taken place or the performed surgery was clearly not suitable to be main factor for fluid collection, the patient was classified into the “non-surgery group”.

### 2.4. Radiation Exposure

In order to estimate the radiation exposure from the additional CT scan, the DLP was acquired from the patients’ CT dose protocol. The DLP was then multiplied with a medium conversion factor of 0.017 mSv/mGy × cm to obtain the effective dose (ED) [21], since this was the average conversion factor for the chest, abdomen and pelvis.

### 2.5. Data Processing

Data were transferred to an Excel spreadsheet (Excel for Mac, Version 15.37, Microsoft, WA, USA) and additionally transferred to the statistical software R (Version 3.4.1, R Foundation for Statistical Computing, Vienna, Austria) for further evaluation. Correlation between the surgery and the non-surgery group was assessed using the adjusted chi-squared statistic proposed by Donner to account for the clustering of the data [22]. A *p*-value of *p* ≤ 0.05 was considered the cut-off point of statistical significance.

## 3. Results

A total of 499 CT guided PADs in 352 patients (123 female, mean age 60 years, range 11–87 years) were identified from our database during the analyzed 33-month period. In 197 of the 499 PADs (39.5%), diluted CM was applied after the CT-guided intervention through the drainage, and a CT scan of the region of interest was performed. We evaluated these 197 PADs in 151 patients. A total of 302 PADs placed in 238 patients without CM application and an additional CT scan after the PAD were excluded. Of these residual 197 PADs, 120 drains had a history of recent surgery (95 patients) and 77 drains (56 patients) had no surgical or other interventions before drainage installation (Table 1).

Considering all included cases, we were able to obtain additional imaging findings due to the contrast flush of the PAD after the intervention in 51 drains (46 patients (25.9%)) with the subsequent detection of 22 fistulas, 13 bile leaks, 12 surgical insufficiencies, and 4 other findings (Table 2).

In 19 drains (19 patients; 9.6%), based on the contrast-flushed PAD CT examination, the therapy was changed based on the contrast-flushed PAD CT examination (Table 3). There was no statistically significant difference between the two subgroups with and without preceding surgery (Table 1).

The average DLP throughout all additional scans after CM injection through the drain was 195.11 mGy × cm. We calculated an effective dose of 3.32 mSv for the additional scan using a medium conversion factor of 0.017 mSv/mGy × cm regardless of sex, age, or body area.

After analyzing all medical reports, there were two reported cases (1%) of SIRS due to bacteremia after CT-guided PAD and the contrast flush of the drain before CT.

## 4. Discussion

CT-guided PAD is a common and frequent radiological interventional method to drain fluid collections within the thorax and abdomen. In the clinical routine, an ultrasound-guided approach is regularly used in more superficial fluid collections, while CT allows a more complex approach to deeper areas in the abdomen and pelvis. Currently, there is no clear evidence-based recommendation of whether a contrast flush with a subsequent CT scan of the drained cavity after the intervention is beneficial for further diagnosis and therapy. There are even concerns of causing SIRS due to the drainage flush [17,18]. This might be one of the reasons why drainage contrasting is not routinely performed.

In our study group we were able to detect 25.9% of additional imaging findings in 197 PADs. These results are concordant with Bélair et al. [13], who reported a detection rate of 32% in patients with fistulas. Furthermore, we were able to initiate 19 immediate changes in therapy, which accounted for 9.6% of the cases. Recently, some studies have focused on the intracavitary application of ultrasound contrast medium in abscess drains. Ingee et al. [15] reported on “additional treatment” in 40% of the cases and in communication with surrounding structures in 14% in 71 patients with intracavitary ultrasound CM application. Especially in abdominal abscesses such fistulas might be hard to detect using ultrasound. Xu et al. published a sensitivity as low as 72% in the detection of post-surgical gastrointestinal fistulas [23]. CT drains of abdominal fluid collections are often performed when ultrasound guidance is not feasible (e.g., for obese patients), and therefore fistulas from the drainages discussed in this publication should be even more difficult to detect.

In this study, we divided our patients into a “surgery-group” and a “non-surgery group”, assuming a difference in the detection of additional findings and expecting the surgery group to show more additional imaging findings due to the contrast flush. However, the two subgroups did not differ, mainly due to the fact that patients from the non-surgery group had a high percentage of Crohn’s disease or acute pancreatitis—two diseases that are very likely to develop fistulas [24,25]. Due to its aggressive nature pancreatic fluid, especially in necrotizing pancreatitis, can develop pancreatico-enteric fistulas in up to 20% of cases [26] (Figure 3). Pancreatitis accounted for five of the six findings that led to an immediate change of therapy in the non-surgery group in this study.

The estimated calculated dose of the post-procedural additional scan resulting in an average of 3.32 mSv in our study was just a coarse estimation based on general conversion factors. In this study, the additional scans were performed with a full dose (120 kV)—low dose scans should be sufficient to detect the contrast medium, especially when a comparison with the pre-procedural scan in the full dose is available to identify anatomic structures [27]. Further studies should emphasize this question.

In our opinion, the type of drainage placed should not play an essential role as to whether a fistula is depictable since the drain is placed anywhere in the fluid collection and not in the fistula itself. However, CMs with higher viscosity should be avoided, in the opinion of the authors.

In this study, roughly 1% of the patients developed SIRS during the procedure. There might be a bias to these data, since inflammatory reactions that developed later (e.g., in the surgery ward) were not covered in the primary radiological documentation. On the other hand, those late onset SIRS might not be directly related to the injection of diluted CM via the drain since SIRS can also develop during the puncturing process, where no CM is injected (e.g., PTBD) [28]. This might be because small vessels are affected by the puncturing of the fluid collection, allowing bacteria to access the bloodstream.

This study was mainly limited by its retrospective nature, its inhomogeneous patient groups, and its single center setup. Further prospective studies are required for further evaluation of the results obtained in this study.

Based on our data, we recommend postprocedural flushing of CT guided drains as a minimal invasive additional CT examination with a very low incidence of major side effects, resulting in relevant additional findings with consecutive changes of therapy in approximately 26% of the patients.

## 5. Conclusions

The postprocedural flushing of CT guided drains with diluted CM provided valuable clinical information in approximately 26% of cases. 

In 10% of patients, a change of therapy was induced by the additional CM application and scan. 

The risk of SIRS during and immediately after the radiological procedure induced by the contrast flush seemed to be significant low (1%). In addition, late-onset SIRS might not be directly related to the CM injection.

## Figures and Tables

**Table 1:** Change of therapy and additional imaging findings based on additional flushing with contrast media (CM) and the patient’s group (with or without prior surgery).

	Drains in Patients with Prior Surgery	Drains in Patients without Prior Surgery	Significance
Drains flushed with CM	120	77	n/a
Additional imaging findings based on CT with CM flushed drainage	32 (26.7%)	19 (24.7%)	*p* = 0.77
Change in therapy based on CT with CM flushed drainage	13 (10.8%)	6 (6.5%)	*p* = 0.38

**Table 2:** Detailed additional findings of the 51 contrast-flushed PADs based on CT imaging.

Additional CT Findings after Contrast-Flushed PAD	Total Number of Findings (*n*)	Number of Findings “Surgical Group” (*n*)	Number of Findings “Non-Surgical Group” (*n*)
Fistulas	22	11	11
Bile leaks	13	8	5
Insufficiencies after surgery	12	12	n/a
Misplaced drains	1	0	1
Descending psoas abscess	1	0	1
Insufficiently drained combed cavity	1	1	0
Contact with aortic prosthesis	1	0	1

**Table 3:** Details of the change of therapy based on CT imaging findings after contrast-flushed PAD.

Change of Therapy Based on CT Imaging Findings after Contrast-Flushed PAD	Change of Therapy (*n*)
PTBD/ERCP	9
Surgery (revision)	3
Change of antibiosis	3
Endoscopic procedure	2
Repositioning of drainage	2

## Data Availability

Not applicable.
